# Youth Participation in Agriculture: A Scoping Review

**DOI:** 10.3390/su13169120

**Published:** 2021-08-14

**Authors:** Wendy Geza, Mjabuliseni Ngidi, Temitope Ojo, Adetoso Adebiyi Adetoro, Rob Slotow, Tafadzwanashe Mabhaudhi

**Affiliations:** 1 Centre for Transformative Agricultural and Food Systems, School of Agricultural, Earth and Environmental Sciences, University of KwaZulu-Natal, Private Bag X01, Scottsville 3209, South Africa; 2 African Centre of Food Security, School of Agricultural, Earth and Environmental Sciences, University of KwaZulu-Natal, Private Bag X01, Scottsville 3209, South Africa; 3 Agricultural Extension and Rural Resources Management, School of Agricultural, Earth and Environmental Sciences, University of KwaZulu-Natal, Private Bag X01, Scottsville 3209, South Africa; 4 Department of Agricultural Economics, Obafemi Awolowo University, Ile-Ife 220282, Nigeria; 5 Disaster Management Training and Education Centre for Africa, University of the Free State, Bloemfontein 9301, South Africa; 6 Centre for Transformative Agricultural and Food Systems, School of Life Sciences, University of KwaZulu-Natal, Private Bag X01, Scottsville 3209, South Africa

**Keywords:** agriculture, capacity building, inequality, policy, poverty, unemployment

## Abstract

Providing economic opportunities for youth in agriculture is essential to securing the future of agriculture in Africa, addressing poverty, unemployment, and inequality. However, barriers limit youth participation in agriculture and the broader food system. This scoping review aimed to investigate the opportunities and challenges for youth in participating in agriculture and the food system in Africa. This review conducted a scoping review using the PRISMA guideline. Published studies were retrieved from online databases (Web of Science, Cab Direct, and Science Direct) for 2009 to 2019. The findings showed that existing agricultural interventions are production-centric and provide low-income earnings and inadequate social protection. We also found that the youth have pessimistic perceptions about agriculture’s capability of improving their living standards. This could be ascribed to the minimal youth involvement in agricultural activities and the youth’s shared understanding of the agricultural sector’s contribution to general economic growth. From a policy perspective, the literature revealed that current agricultural development programs do not adequately address structural issues underpinning youth participation in the economy. Therefore, to enhance the involvement of youths in agriculture, there is a need for policy implementation in the area of integrated agricultural-based interventions that are context-specific and promote meaningful youth participation in shaping future food systems.

## 1 Introduction

Youth unemployment is a challenge in the global south [1]; in particular, Africa faces significant challenging conditions, similar to Asia’s in previous decades [2]. This is primarily due to rapid population growth, slow economic growth, a higher unemployment rate concomitant with a large volume of unskilled workers, and an ageing and declining agricultural sector [3]. Sub-Saharan Africa (SSA) displays the highest poverty rates among youth, constituting more than 30% of the region’s population [4]. With approximately 60% of Africa’s population under 35 years old, most of its youth are unemployed and living in rural areas [5]. At the current population growth rate, nearly 420 million young people are aged 15–35, with an estimated 10–12 million people joining the labour force and needing new jobs annually [4]. The transition from school into the workplace is becoming increasingly challenging for young people, especially those living in developing countries; three out of four find employment in the informal sector [6].

Agriculture is recognised as a primary livelihood source for many rural people in Africa and an essential contributor to economic growth [7]. Previous research studies [4,5,8,9] and policies [10,11] have highlighted the role of agriculture in employment creation, food and nutrition security, and reducing societal inequality and poverty in Africa. The agricultural sector also presents opportunities for entrepreneurship, which would be ideal for employment creation, especially among youth [12]. Moreover, the increasing growth in Africa’s urban markets further presents an opportunity for increased demand for processed and prepared foods. Consequently, this would attract substantial private sector investments for small, medium, and large agribusiness entrepreneurs and other food system participants. Fulfilling the potential of African agribusiness could open up markets estimated to be worth more than US$100 billion per year by 2025 [13].

Conversely to the urban sector, entrepreneurship within the agriculture sector presents several challenges. For youth, these challenges include poor and/or limited infrastructure [1,14], and a lack of access to finance, production inputs and resources, markets, extension services, and training [15,16]. Additionally, youths are seen competing with older and more established farmers for resources [17]. The youth’s socioeconomic environment mainly portrays farming as a “poor man’s” occupation, characterised by long working hours with low economic returns and social status [18].

Defining the term ‘youth participation’ is essential to understand the link between youth and agricultural development. According to Checkoway [19], youth participation is the active engagement and influence of young people. This is not only based on their passive presence or token roles in adult agencies, but also on quality, such as when people have a real effect on the process, influence a particular decision, or produce a favourable outcome. Furthermore, youth participation assumes that young people are competent citizens rather than passive recipients of services. Finally, it involves young people in the institutions and decisions that affect their lives. Therefore, youth participation in agriculture entails the engagement of youth in the sector through entrepreneurial activities, participation in value-chain activities, policy formulation, and advocacy in structures and systems linked to the food system.

While youth participation is vital for the growth of a nation’s economy, youths also face additional socio-economic barriers that underpin their involvement in the agricultural sector. Such barriers include parents’ discouragement of youths from pursuing careers in farming [18] and opting for white-collar professions [20] that are thought to have higher economic returns and fewer risk factors [21]. Additionally, for most youths in rural areas, the choice to be involved in farming is determined by their immediate need to fulfil basic needs, lack of employment opportunities, or assurance to inherit the land [22]. When it happens, participation is circumstantial instead of being aspirational; the youth will often leave in pursuit of a “better life” in urban areas when opportunities present themselves [16,20].

A growing body of knowledge suggests that part of the solution for promoting youth participation in agriculture should include supportive policies and frameworks that promote capacity building [20,23], stakeholder investment, and creating innovative spaces in agriculture in a way that takes into account youth aspirations. Such policies and frameworks should be inclusive and recognise at the onset young people as key players in policy formulation [10,24]. However, this should be complemented by other initiatives as well. In a policy analysis conducted on agricultural, rural development, youth and employment policies in SSA, Schwebel et al. [25] concluded that policies focus more on promoting labour supply strategies, such as training programmes on entrepreneurship skills, rather than the strategies on the demand side, such as reducing the constraints to business development and job creation at the sectoral level. Moreover, this focus fails to respond to the region’s human capital shortcomings, which result from poor-quality education and a lack of employable skills. Thus, upgrading the skills of young people is essential in ensuring their participation in agriculture, promotion of food security, and reducing poverty in rural, peri-urban, and urban areas [26].

Furthermore, the nature of the workplace is continuously changing, and technological advancements are altering labour market requirements. The combination of these transformations, increasing inequalities, and economic stagnation makes it more difficult for all individuals to find decent work opportunities. As a result, it is essential to prepare and equip young people with the necessary skills, education, ambition, and aspiration to be employable [6]. Therefore, the focus should shift to thinking about the kind of action needed for youths to take on more active roles in society [27], removing obstacles for youth engagement, and creating an enabling environment for young people to thrive [23,28,29]. Africa has identified agriculture as a sector that could drive such socio-economic transformation and allow for broader participation of marginalised groups, women, and youth, in the economy. However, it is not clear whether the intent has translated into the desired outcomes. This scoping review, therefore, aimed to assess the opportunities and challenges for youth participation in agriculture and the food system in Africa. In this paper, the primary hypothesis is that there is limited understanding of youth’s perception of their role in the food system and the economy and that production-centric policies limit effective youth participation in the agricultural value chain and food system.

The specific objectives were to:

(i) Identify the existing challenges and opportunities for youth in agriculture in Africa;
(ii) Based on the evidence, propose an integrated agricultural-based approach for promoting youth participation and inclusivity in agriculture and the future food system in Africa.

## 2 Materials and Methods

### 2.1 Literature Search

**Figure 1:**
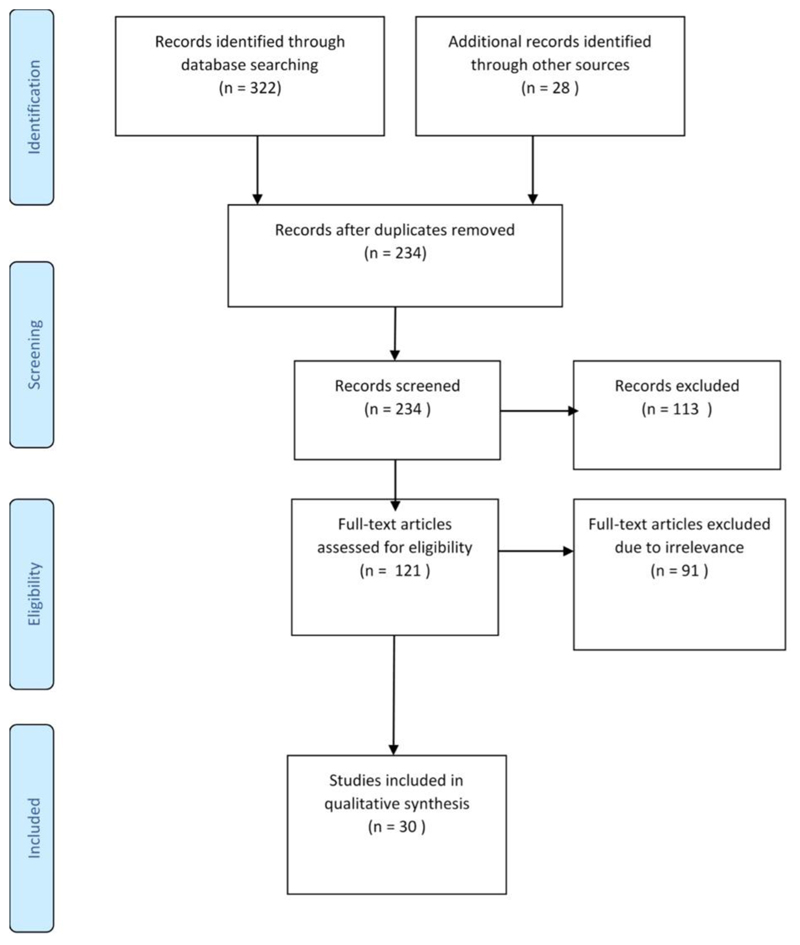
PRISMA flow diagram outlining protocol adopted in the scoping review based on the Preferred Reporting Items for systematic review and meta-analysis protocols (PRISMA-P) 2015 statement Moher et al. [31].

The Preferred Reporting Items for Systematic Reviews and Meta-Analyses checklist was used as a guideline to avoid biased reporting [30–32]. The PRISMA flowchart (Figure 1) was used as a guideline for reporting the review results. The literature search included peer-reviewed research articles using online databases, namely, Web of Science, Cab Direct, and Science Direct, based on studies on youth in agriculture in Africa published between 2009 and 2019. The period was chosen because it corresponds to the African Youth Decade plan of action [10]. Grey literature and relevant policy documents (regional policies set by international government organisations obtained from websites of key development organisations with known involvement in agriculture in Africa such as the Food and Agriculture Organization (FAO), African Union, Southern African Development Community (SADC), New Partnership for Africa’s Development [NEPAD], Institute of Development Studies, and the United Nations, among others).

The PCC (population, context, and concept) nomenclature was adopted to determine the eligibility of this study’s research question for a scoping review, and to determine eligibility criteria for the reports included. In terms of population, the study included young people between the ages of 15 and 35, as defined by the African Youth Charter [33]. Aside from agriculture involvement, the context was also inclusive of young people in universities/agricultural institutions or any other agricultural training program. The literature search terms/keywords used were ‘agriculture’ with the synonyms’ farming’, ‘land management’, and ‘farm management’. The second keyword used was ‘participation’ with the synonyms’ involvement’, ‘engagement’, and ‘contribution’. The third keyword used was ‘youth’ with the synonyms’ young people’, ‘adolescents’, and ‘young adulthood’.

The keywords were used in combination with each other. The use of singular, plural, and synonyms for search terms was also applied, accounting for relevant keywords that may differ from one database to another. For example, for the search term ‘youth and agriculture’, ‘young farmers’ or ‘young people in farming’ was used. Articles were considered for the review if the keywords appeared either in the title, abstract, or were discussed as a heading/subheading.

The full screening of records retrieved focused on articles that reported on one or more of the following outcomes concerning youth: agriculture interventions, policies, opportunities and challenges, food systems, livelihoods, development, and career aspirations in agriculture or agribusiness. Additionally, there was a focus on studies researching youth perceptions, awareness, or attitudes towards agriculture, program, or intervention analysing the economic, social, or political environment factors affecting the participation of young people in agriculture, studies conducting research/addressing a challenge or limitation faced by youth in agriculture and rural youth livelihood strategy studies. Included studies were those that were conducted in Africa or of relevance to Africa.

Studies on food security, diets, nutritional status, farm injuries in young workers, and urban youth migration, gender gaps and their influence on youth challenges or opportunities and literature reviews were excluded due to differences in context and associated context-specific challenges and opportunities. A total of 322 studies were retrieved from online databases (Table S1) and 28 records from other sources (Table S2). After a comprehensive search and screening of titles, 116 duplicates were removed, and 234 abstracts were screened for eligibility. A total of 118 studies were excluded as they were found to be unrelated to the study’s objective. The full-text articles of the remaining 121 articles were downloaded for further screening. During the full article screening stage, 91 of the 121 studies were also excluded. Some of the reasons for exclusion were based on study type (review paper, opinion pieces and policy briefs with no primary data), variables being investigated not being aligned with the inclusion criteria, and the research being conducted outside of Africa (see Supplementary Information Table S3). A total of 30 articles met the selection criteria and were included in the study (refer to Figure 1 above). These articles include six policy documents that met the selection criteria. These six policy documents were then further reviewed based on youth inclusion as part of the specific policy objectives.

### 2.2 Data Analyses

The 30 selected articles were imported into the QSR NVivo 12 [34] qualitative data analysis software. The first objective was achieved by conducting a search query for the 20 most common words in the data set, and word trees were generated. The number 20 was chosen to give a snapshot of broad focused themes and connections within the data set related to opportunities and challenges for youth participation in agriculture. These were then translated into themes or ‘nodes’ for further analysis [34]. The nodes consisted of classifications such as ‘development challenges’, ‘demographical challenges’, ‘unemployment’, ‘youth perceptions and aspirations’, ‘youth characteristics’, ‘youth participation’, ‘development programs’, and ‘policy priorities’. Further classification of the data set was carried out to code passages of the data under the appropriate node. An analysis was conducted using ‘matrix queries’ and ‘cross-tabulate functions’ within the software to establish hierarchal relationships between nodes and link similarities between the data to achieve the second research objective. A query was also conducted to extract qualitative and quantitative data on youth participation, perceptions, interest, and aspirations from the data set. Using functions within NVivo, aspects of the data set and results from the first two objectives were further analysed for patterns and casual relationships and visualised using maps and diagrams to determine the role of youth in future agricultural food systems in Africa, thus achieving the third study objective.

## 3 Results

### 3.1 Literature Search

A total of 24 studies and six policy documents were included in this review. The six policy documents that met the inclusion criteria are: (i) African Youth Decade Plan of Action 2009–2018, (ii) African Agenda 2063, (iii) African Youth Charter, (iv) Comprehensive Africa Agriculture Development Programme (CAADP), (v) the Malabo Declaration on Accelerated Agricultural Growth and Transformation for Shared Prosperity and Improved Livelihoods, and (vi) Regional Agricultural Policy (RAP).

A total of 91 studies were excluded at the full-text screening stage (see supplementary information Table S3). The most common reasons for exclusion included: no primary data or clear description of the age range of youth (specifying age range remains an important selection criterion [35–37], variables being investigated by the study were not aligned with the inclusion criteria, research was conducted outside of Africa, and the results are not relevant to Africa, opinion pieces based on a workshop, the article had been removed from the website, and there was no English version of the article available.

The characteristics of included studies are presented in Table S4 (Supplementary Information). The majority (44%) of the included published studies were conducted in the West Africa region, primarily Nigeria, followed by the SADC region (16%). Most of the studies had a focus on rural areas. The studies investigated youth involvement in agriculture from various perspectives, such as the adoption of production technologies by youth [38], challenges for youth in agriculture [23,39–42], policy engagement [20,24,29], youth perceptions, career goals and aspirations, entrepreneurship opportunities [15], and information communication and technology [28]. Most studies (n = 13, 54, 2%) did not mention or refer to any policies related to youth or youth participation in agriculture. Eleven studies mentioned policies, as shown in Table S5 (see Supplementary Information).

### 3.2 Challenges and Opportunities for Youth in Agriculture

The studies identified by the review were unable to articulate the key priority areas for youth in agriculture. However, some studies suggested improvements in the rural environment to encourage youth participation in agriculture. These improvements include the incorporation of youth aspirations in agriculture [20,22], capacity building and the development of rural infrastructure [23], improving the image of agriculture [18,28,41,43], and the engagement of youth in policy processes [24].

**Figure 2:**
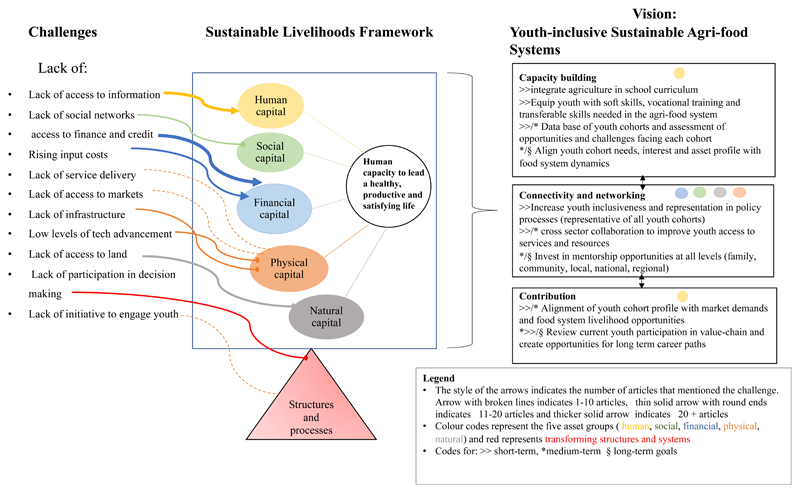
Conceptual framework for promoting youth inclusivity and meaningful participation in agriculture and the food system. Adapted from Mabhaudhi et al. [44].

Challenges that affect youth participation in agriculture were discussed and analysed in most studies (n = 13, 54, 2%). Figure 2 highlights the number of studies across each challenge. The density of the included studies across each challenge is mapped onto the conceptual framework and has been integrated into identifying key pathways for the role of youth in future agricultural food systems in Africa. These challenges were mostly related to access factors (production resources, finance, knowledge and information, extension, innovation, and technology) and control over resources. Lack of education, career guidance, employable skills to enter the job market, mentorship, resources, and supportive policies were mentioned as other challenges affecting youth participation in the economy. Using the sustainable livelihood approach, physical capital challenges such as service delivery, access to markets, infrastructure, and low technological advancements were the most dominant. Human and financial capital, that is, access to information and finance and credit, respectively, were mentioned as a challenge across most articles. It was interesting to note that social capital was mentioned as a challenge. Youth in agriculture have embraced information and communication technology (ICT) and use different networking platforms such as Facebook, Google, and WhatsApp. Unfortunately, the cost of data has prohibited the full use of ICT for networking.

At a regional level, the importance of investing in youth empowerment and development is mentioned in most of the policies (see Table 1). The main challenges mentioned by the policies are an inadequate investment in youth development, lack of youth socioeconomic empowerment, provision of essential services, and a lack of education. However, there is no exclusive mention of the role of youth in agri-food systems. Youth, women, and ‘other vulnerable members of society’ are mentioned as beneficiaries of what the projected ‘agricultural development’ will yield [11,45,46].

### 3.3 Youth Aspirations, Interest and Participation in Agriculture

Only four studies (16%) [15,18,22,47] identified youth interest and career aspirations, which were mainly corporate careers or ‘modern jobs’. Only youths involved in agriculture as a primary occupation, members of agricultural clubs/organisations or participants of agriculture-related interventions (for example, the Young Farmer’s Club from Nigeria [47]) had aspirations of choosing agriculture as a career.

Youth participation and pursuing agriculture as a career was mentioned in terms of negative or positive experiences and perceptions of high school students, graduates, and rural youth. Adverse experiences were associated with demographical challenges related to marital status, education levels, access to resources, and information in rural areas [20,36,40,47–50]. A demographical challenge was the retention of land by the elderly [16,20,22,23,51], where land in rural areas is used as a bargaining chip for power and respect. Young people gain access to land through inheritance, permission to occupy agreements, or leasing [39].

Some studies [18,39] highlighted that elderly people perceive the youth as self-centred, ‘problematic members’ in society, who are ‘inexperienced’, only concerned about their economic welfare and their own immediate family, rather than extended family needs. These perceptions negatively affect youth regarding accessing land for agricultural use, as they would then not be prioritised. Additionally, when it comes to land allocation, preference is often given to married youths [51]. These experiences directly influence how youths perceive agriculture, their interest in participation, and their career aspirations. Positive perceptions of agriculture are related to youth experiences of participating in agricultural programs, the influence of family members who are farmers, and access to services and resources. These experiences were also linked to the entrepreneurship opportunities that youth perceived agriculture to have, making them want to pursue it as a career.

Youths who are already involved in agriculture through participation or exposure to training programs are more likely to have future aspirations related to agriculture than those who are not. Additionally, socio-economic factors such as the youth’s marital status, education levels, access to resources and information, and perceptions of community resource ‘gatekeepers’ about youth influences how youths perceive agriculture and their interest in participating.

### 3.4 Role of Youth in the Food System

Based on the literature search, most youths were involved in primary activities such as crop production (for example, cassava, cocoa, rice, maize vegetable production) and animal production (for example, livestock rearing, breeding, poultry, and poultry piggery). An analysis of the value chain showed that very few youths participated in mid-point activities such as agro-processing and agricultural engineering. One study was conducted on youth involvement in fish farming [52]. In eight studies, youths were involved in agriculture as their primary occupation, followed by trading activities and government jobs. The remaining youth were students, and others were involved in other livelihood activities such as trading and other forms of temporary employment. The majority of the studies did not mention any ownership of land by the youths. However, in studies where it was mentioned, the average farm sizes owned by youths ranged from 1.5 to 3 ha [39,43,48,49,53]. Additionally, there was no mention in any study of youth involvement in dealing with issues concerning the food environment.

In summary, youth participation in agriculture is mainly concentrated in the primary sector. Youths who have access to land have plots that are less than 3 ha. Furthermore, there is little youth participation in value-chain activities, and no evidence of youth advocacy in structures governing issues related to the food system.

## 4 Discussion

This scoping review identified various areas of existing research on youth involvement in African agriculture. The key challenges for youth participation in agriculture are centred around knowledge availability; production resources; and lack of infrastructure, support, and access to advisory services. The findings suggest that building the capacity of young people is essential in ensuring their participation in agriculture. Yeboah and Jayne [21] noted that although the number of people employed in agriculture is still increasing, the workforce share is declining over time. This suggests that young people are fleeing from poverty and not farming per se. The majority of youths in SSA reside in rural areas, where labour productivity is low and employment opportunities are scarce [54]. With farming generating about 68% of rural income in Africa [55], youths are more likely to find employment in the informal sector or engage in self-employment to meet immediate basic needs [56].

However, underemployment offers limited social protection and rights, low wages, poor job security, and limits future career development opportunities [57], perpetuating the cycle of poverty and hunger. Therefore, addressing youth participation in agriculture requires a holistic approach, broadly focused on improving rural economies, social well-being, and service delivery [26]. Moreover, making farming more profitable and less laborious could potentially attract youth into agriculture [21].

In this paper, we used the results from the review on challenges faced by youth in agriculture, youth aspirations, interest, youth participation in agriculture, and the sustainable livelihoods (SL) framework to shed light on the micro-dynamics that embody youth capabilities, their productivity, enabling environment, outcomes of livelihood strategies, and ultimately their contribution in society [58]. A framework (see Figure 2) was developed to map key pathways for the role of youth in future agricultural food systems in Africa. The human development perspective and the capability approach highlight the importance of enhancing people’s abilities to lead productive and fulfilling lives, which they value [59,60]. Consequently, stressing the importance of investing in well-being, expanding people’s choices, empowerment, and removing obstacles preventing people from actively participating in life.

For youth to participate in food futures, aspirational career paths with a long-term perspective other than economic gains or reducing unemployment would have to be created locally, nationally, and globally [55]. However, the challenges faced by youth in agriculture (see Figure 2) affect all aspects of their capital assets, which puts an additional constraint to their baseline, regardless of the presence, or lack thereof, of an enabling environment for youth to succeed in the sector. Additionally, these challenges also come from a lack of involvement in policies and institutions governing youths’ surroundings [20,24]. These factors alone limit youth inclusivity and any prospects of youth positively contributing to agriculture and society.

The interactions between the physical, social, environmental, and institutional factors play a role in individuals’ overall capacity and capabilities [61,62]. Therefore, investments made should be balanced and well distributed amongst these aspects for a more significant impact. Addressing youth participation in agriculture requires a holistic approach, broadly focused on improving rural economies and creating employment opportunities for youth. As long as rural areas suffer from a lack of service delivery, poor connectivity, and stagnant economies, it will be difficult to convince anyone to stay there [7,63]. Furthermore, there is the potential for job creation for young people in non-farm activities related to food value chains, sustainable agriculture, agribusiness, and other food system support services. However, unlocking these economic opportunities relies on strengthening rural–urban linkages [13].

For youths to effectively contribute to agriculture, investment must be made to develop their capacity by investing in human capital (for example, education, soft skills, vocational training, and skills development). There is a link between the level of education attained and youth participation in agriculture. As noted by Bezu and Holden [64], education improves youths’ access to information about opportunities outside of their immediate surroundings, thus raising expectations and encouraging youths to explore these opportunities. Youths who have low levels of education resort to agriculture as a means of earning an income, as they do not meet the requirements for employment in other professions [65].

On the contrary, McMillan and Harttgen [66] note that three out of five young workers in sub-Saharan Africa do not have the level of education required to make them productive in the workplace. Additionally, only 2% of African university graduates specialise in agriculture, with nearly 80% of young people aged 25–34 working in agriculture having a primary school education or less, including 40% with no education at all. The inclusion of agricultural subjects and activities in schools could spark an interest in young people and expose them to various aspirational career opportunities in agriculture at a young age [67]. Similarly, Magagula and Tsvakirai [68] found that exposure to agricultural studies at secondary and tertiary levels influences youths’ intention to participate in agripreneurship. This highlights the importance of promoting agricultural education at the different levels of education, i.e., primary, secondary, and tertiary, as part of the overall strategy to increase awareness on agriculture as a career choice and hence increase youth participation in agriculture.

Secondly, youth’s connectivity to society, industry role players, and stakeholders, and their access to resources and services, must be enhanced through investments in social, financial, natural, and physical capital, particularly basic infrastructure and service delivery in rural areas (secure shelter and buildings, water supply and sanitation, health, energy, communications). The challenges due to the lack of general service delivery in rural areas hinder young people’s engagement and success in agriculture [69]. They may be the reason behind their low participation and negative perception of agriculture. Undoubtedly, the challenges associated with young people’s access to productive resources and services in rural areas also contribute to low participation. For example, the customary laws and norms associated with accessing land often discourage young people from viewing agriculture as a livelihood option. Facilitating dialogue and connection between youths (representative of all socioeconomic backgrounds), the private sector and industry players can be valuable for enhancing youth’s connectivity and inclusiveness to society and the sector.

Building the capacity of youths through education and other initiatives and leveraging that to improving their socioeconomic access to industry players in the agricultural sector will increase the likelihood of youth engagement in farming activities [33,70]. As a result, young people would be empowered while gaining the necessary skills to participate in evolving global agriculture value-chains [69]. Moreover, how millennials connect to the food system is determined by the biophysical, economic, cultural, and social domains, shaping how they perceive themselves actively contributing to the food system [71]. It is therefore imperative for policies, strategies, and interventions targeted at promoting agricultural growth, development, and participation of youths in the sector to speak to the socioeconomic context, needs, goals, and aspirations of youths [33].

## 5 Limitations

The results of the study should be considered in light of some limitations. Due to the search inclusion and exclusion criteria used for this study, other publications may be excluded. The search was limited to studies published between 2009 and 2019, as 2009–2018 were the implementation years for the African Youth Decade plan of action [10]. Furthermore, the selection of primary studies focused on programs or interventions that have been explicitly designed for youth, between the ages of 15 and 35 years. Thus, the data presented in this study are from papers relevant to the study’s objectives. For future research, we encourage broadening the scope of the research to ensure the inclusion of the full spectrum of themes related to youth and agriculture; however, this should take into consideration the trade-offs between breadth vs. depth.

## 6 Way Forward: The Role of Youth in Future Food Systems

Based on evidence found in this scoping review, this study therefore recommends:

- The exposure to agricultural studies at secondary and tertiary level could influence youth’s intention to participate in agripreneurship. This will expose youths to the full range of career options in the agricultural sector at earlier stages. This is being carried out in Zambia through the UniBRAIN program by promoting agricultural innovation and improving tertiary agribusiness education in Africa. In South Africa, the Junior LandCare programme creates an environment for school children to participate in sustainable agriculture activities through food gardens that supplement the school feeding scheme.
- National agricultural policies need to account for youth aspirations, contributing factors, challenges facing youth under different contexts, and characterise data based on age groups. This will assist in developing youth tailored initiatives relevant to the context.
- For youths who are inclined to work in agriculture, high-potential value chains that align with their aspirations and have the potential for increased economic returns should be identified, including the provision of support and removing barriers for youth participation, for example, the YEAP program in Nigeria and Youth Agropastoral Entrepreneurship Programme in Cameroon. These initiatives are creating decent employment opportunities along the agricultural value chain for youths in rural areas. Initiatives can be facilitated through the implementation of regional policies such as the Malabo declaration and Agenda 2063.
- For youths who are not inclined to work in the farm, including those without access to land and production resources, mobilization of support through government and the private sector could position these youths in nonfarm activities that drive agricultural transformation and the improvement of rural markets. For example, marketing and trading of agricultural related products.
- Regional strategies for developing the agricultural value chain (for example the SADC Regional Agricultural Policy) must mainstream youth considerations, with the objective of youth inclusion, capacity development, and sustainable employment opportunities. There is a need for more deliberate investments to be made to create opportunities for youth throughout the value chain.
- Efforts must be made to create a supportive environment to increase opportunities through which youths can pursue food system-related careers and interests. This can be achieved through increasing social capital, improving youths’ connectivity to value-chain actors, promoting networking, peer-to-peer learning, raising awareness, mentorship, and other forms of linkages.
- Modernisation of agricultural production systems and promoting the development of local value-chains to increase awareness, provoke youth interest, and establish relevant role models. Additionally, the design of national policies should explicitly support informal businesses in rural and peri-urban areas.

## 7 Conclusions

The study sought to investigate youth participation in agriculture in Africa by mapping evidence around policies, opportunities, and challenges for youth to pursue a career or be engaged in agriculture. This was carried out through a scoping review using the PRISMA extension and published studies retrieved from online databases (Web of Science, Cab Direct, and Science Direct) for 2009 to 2019. The findings showed that existing agriculture interventions are production-centric, provide low-income earnings, and inadequate social protection. Without deliberate investments in creating opportunities for youths in the food system, the role of youth will continue being primarily concentrated in the primary sector leaving other aspects of the value-chain untapped. The inclusion of agricultural subjects and activities in schools could spark an interest in young people, and also expose them to a variety of aspirational career opportunities in agriculture at a young age. Although there has been a growing body of knowledge in youth participation in agriculture in the past few years, the literature is scattered with limited coherence. We also found that the youth have weak perceptions about agriculture’s capability of improving their living standards. This could be ascribed to the minimal youth involvement in agricultural activities and youths’ low understanding of the agricultural sector’s contribution to general economic growth. From a policy perspective, the literature revealed that current agricultural development programs do not adequately address structural issues underpinning youth participation in the economy. Therefore, to enhance the participation of youths in agriculture, policy interventions should adopt a value-chain and food-systems approach to open up more opportunities for youths in the sector. Moreover, these policies should be supported by policies aimed at increasing access to finance/credit and broad capacity development.

Some countries in Africa have begun implementing programs and initiatives to increase youth involvement in agriculture. This is being carried out through skills development, facilitating access to resources, promoting agricultural innovation and integrating agribusiness into tertiary education. Such interventions can increase innovativeness among youth and attract them toward agribusiness. However, the level of investment remains low, with most interventions not responding to the dynamism of youth. There needs to be an account for youth aspirations, contributing factors, challenges facing youth under different contexts. This will assist in developing youth-led initiatives that take into consideration their needs and are relevant to the context.

## Supplementary Material

Supplementary Information

## Figures and Tables

**Table 1:** The inclusion of the ‘youth agenda’ in policy documents selected for the review.

Policy	Challenges Faced by Youth	Youth Role and Participation in Agriculture	Youth Investment Focus Areas
African Youth Charter	Not mentioned	Not mentioned	▪ Provision of access to health care ▪ Guaranteed participation of young people in government and spheres of society ▪ Provision of technical and financial support to build the institutional capacity of youth organisations ▪ Provision of access to information and services which will empower youth to become aware of their rights and responsibilities ▪ Education and skills development ▪ Poverty eradication and socioeconomic integration of youth ▪ Sustainable livelihoods and youth employment
African Youth Decade Plan of Action	▪ Unemployment, underemployment ▪ lack of skills, relevant education, access to capital, unmet need for health-related information and services	Not mentioned	▪ Ensuring a rights-based approach to youth development through meaningful participation and representation ▪ Consolidated investment targeting youth socioeconomic empowerment ▪ Mainstreaming youth perspective in the efforts to achieve broad development goals and priorities ▪ Investing in youth empowerment and development ▪ Investing in meaningful participation and contribution of young people towards Africa’s progress and sustenance of current gains. ▪ Advocating for the well-being of youth by having access to education, health facilities, employment and promoting the cause of the disadvantaged youth.
Agenda 2063	▪ Unemployment and underemployment ▪ Access to education, training, skills and technology, health services, jobs and economic opportunities,	Not mentioned	▪ Inclusive growth, job creation, increasing agricultural production; investments in science, technology, research and innovation; gender equality, youth empowerment and the provision of basic services including health, nutrition, education, shelter, water and sanitation ▪ Engagement and empowerment of youth through the full implementation of the African Youth Charter ▪ Youth issues mainstreamed in all development agendas. ▪ Africa’s youth shall be the driving force behind the continent’s political, social, cultural and economic transformation.
CAADP	Not mentioned	Not mentioned	Not mentioned
Malabo Declaration	Not mentioned	Not mentioned	▪ To create job opportunities for at least 30% of the youth in agricultural value chains ▪ To support and facilitate preferential entry and participation for women and youth in gainful and attractive agri-business opportunities.
SADC Regional Agricultural Policy	youth is poor, rural, has poor access to economic activities, education, land and capital.	Participation for most of the rural youth in farming is based on circumstances and limited economic opportunities	▪ Promoting land policy research and development considering gender, youth and vulnerable groups ▪ Facilitate Member States in promoting agriculture as an attractive career choice for the youth ▪ Facilitating the participation of informal traders, SMEs and marginalised groups such as women and youth. ▪ Mainstream youth needs in regional and national policies and strategies dealing with access to land, farm support systems and services and rural finance.

## Data Availability

The data presented in this study are available on request from the corresponding author.
