# Supplementary material for: Youth Participation in Agriculture: A Scoping Review
Source: Sustainability. Author manuscript; Available in PMC 2023 Sep 11. (PMC7615079; doi:10.3390/su13169120)
Supplement: Supplementary Information [file EMS187376-supplement-Supplementary_Information.docx]

# Scoping review supplementary information

Table Si 1: Scoping review screening records

| **Date** | **Database** | **keywords** | **retrieved articles** | **eligible articles** |
| --- | --- | --- | --- | --- |
| 29/05/19 | Web of science | Agriculture and youth | 84 | 33 |
|  |  | Youth participation OR engagement in agriculture | 838 | 7 |
|  |  | youth opportunities OR prospects in agriculture | 252 | 4 |
|  |  | Youth, agriculture AND limitations | 61 | 2 |
|  |  | Youth and farming | 43 | 7 |
|  |  | youth AND farming AND limitations OR challenges | 101 247 | 0 |
| **sub-total** | | **53** | | |
| 29/95/19 | Cab direct | Youth AND agriculture | 6 | 6 |
|  |  | Youth AND agriculture AND participation AND engagement | 0 | 0 |
|  |  | youth opportunities OR prospects in agriculture | 4526 | 73 |
|  |  | Youth, agriculture AND limitations | 17 | 4 |
|  |  | Youth AND agriculture AND challenges | 150 | 45 |
|  |  | Youth AND agriculture | 1028 | 120 |
| **Sub-total** | | **248** | | |
| 30/05/19 | Science direct | Agriculture and youth | 62 | 6 |
|  |  | Youth AND agriculture AND participation AND engagement | no results found | |
|  |  | Youth AND agriculture AND participation | 4 | 0 |
|  |  | youth opportunities OR prospects in agriculture | 584 | 7 |
|  |  | Youth, agriculture AND limitations | no results found | |
|  |  | opportunities for youth in agriculture | no results found | |
|  |  | Youth AND agriculture AND challenges | 8 | 3 |
|  |  | youth AND farming | 17 | 4 |
|  |  | youth AND farming challenges | 6 | 1 |
|  |  | youth AND farming opportunities | 4 | 0 |
| **Sub-total** | | **21** | | |
| **Total** | | **322** | | |
| 04/06/19 | **after removal of duplicates** | | **206** | |

Table Si 2: Grey literature and policy documents included in the review

| **GREY LITERATURE** | | | | |
| --- | --- | --- | --- | --- |
| **Date** | **Website/ organization** | **Search terms used** | **Documents found** | **Documents considered for review** |
| 03/05/19 | https://au.int/en | "youth and agriculture." | 4 | - A.U. commission strategic plan 2014-2017 - African youth decade plan of action 2009-2018 - Agenda 2063 - Africa agenda for rural transformation |
| 03/05/19 | http://www.fao.org/ | "youth and agriculture." | 6 | - African youth in agriculture - Developing knowledge, skills and talent for youth to further food security and nutrition - Rural youth mobility project - Young and unemployed in sub-Saharan Africa - Youth and agriculture - Youth employment promotion |
| 03/05/19 | http://www.nepad.org | "youth in agriculture." | 7 | - CAADP country implementation under Malabo declaration - African solidarity trust fund - Agricultural education and skills framework - African climate-smart agriculture alliance - Introducing CAADP - Malabo declaration - Sustaining the CAADP momentum to spur agriculture transformation |
| 03/05/19 | https://www.ids.ac.uk | "youth in agriculture." | 8 | - Africa’s youth employment challenge - Agriculture and the generation problem: rural youth, employment and the future of farming - Perceptions and aspirations: A case study of young people in Ghana’s cocoa sector - Stimulating agribusiness entrepreneurship to solve youth unemployment in Kenya - The new alliance on food security and nutrition: What are the implications for Africa’s youth - Young People, Agriculture, and Transformation in Rural Africa: An “Opportunity Space” Approach - Young People in African (Agricultural) Policy Processes? What Can National Youth Policies Tell Us? - Youth, agriculture and land grabs in Malawi. |
| 03/05/19 | https://www.sadc.int | "youth in agriculture." | 3 | - Charter establishing CCARDESA - Regional agriculture policy - SADC business plan on orphans, vulnerable children and youth 200-2015 |

Table Si 3: Reasons for exclusion for articles removed at the full screening stage

| **Author & date** | **Title** | **Reasons for exclusion** |
| --- | --- | --- |
| Muchira, J. (2018) | Stimulating agribusiness entrepreneurship to solve youth unemployment in Kenya | The articles are short policy briefs aimed at providing an analysis and recommendations for policymakers on development issues. It has no primary data or clear description of the age range for youth being referred. |
| Mutua, E. (2017) | Increasing youth participation in livestock production in Kenya |  |
| Naouri, M. (2015) | Mobility of rural youth and new forms of Saharan agriculture (Biskra, Algeria) | The study is not available in English. Full article available written in French. |
| Njeru, L. K. et al. (2015) | Influence of Kenyan youth's perception towards agriculture and necessary interventions; A review | Review paper-no primary data. |
| Ouendeno, M. L. et al. (2015) | Professional trajectories of youth in Saharan neo-agriculture (Biskra, Algeria) re-examined with the agricultural ladder theory. | The study is not available in English. Full article available written in French. |
| Rukuni, M. & Zvavanyange, R. (2014) | Re-configuring the future of Africa's workforce in agriculture and food systems with youth in mind | Policy brief article. No primary data or clear description of the age range of youth being referred to. |
| Sakketa, T. G. & Gerber, N. (2017) | Rural shadow wages and youth agricultural labour supply in Ethiopia: evidence from farm panel data | The study analyses employment trends of youth on and off the farm in Ethiopia, based on secondary research from a consensus. The variables being investigated are not aligned with the inclusion criteria. |
| Soelen, R. W. van & Hoolwerff, R. van (2018) | Youth employment in agriculture through business development and education in Liberia and Mali | Briefcase studies of two young people, with no in-depth information on youth demographics or the age of the participants. |
| Sumberg, J. et al. (2012) | The young people and agriculture 'problem' in Africa | Discussion paper based on a conference themed 'young people, farming and food in Accra'. There is no primary data. |
| Miller, M. E. & B.C Lee (2014) | Developing a Model Policy on Youth Employment in Agriculture | The article is about developing a model policy document for adolescent workers, which includes safety measures, age-appropriate assignments, minimizing injuries and supervision, which is not relevant to the objectives of the research. |
| Makor, J. C. D. & Garbo, M. F. (2014) | The Liberian youth in agriculture | No primary data and no clear aims or objectives of the paper |
| Luckey, A. N.  et al. (2013) | Assessing youth perceptions and knowledge of agriculture: the impact of participating in an AgVenture program | The research was conducted in America, and the results are not relevant to Africa. |
| Kokanova, N. (2014) | The potential of agriculture to create a positive job outlook for rural youth in Africa | No primary data. The paper is a discussion contributing to the broader literature. |
| Khatir, A. et.al (2017) | Factors influencing migration intention of rural youth members/non-members of Marvdasht county Agricultural Cooperatives | The article is written in Persian. English version not available. |
| Jayne, T. S. et al. (2016) | Agri-food systems and youth livelihoods in Sub-Saharan Africa | No primary data. The paper is a discussion contributing to the broader literature. |
| Iyanda, B. (2014) | International policy frameworks: the need to engage Africa's youth in agriculture and natural resources management and its policy development processes | Policy brief discussion article. No primary data, discussion aims, no objectives or clear description of the age range of youth being referred to. |
| Hosaena et al. (2018) | Role of land access in youth migration and youth employment decisions: empirical evidence from rural Nigeria | Review of literature related to the drivers of migration, employment decisions and the role of land access in the choice of livelihood strategies for young people. No primary data. |
| Geerardyn, T. (2018) | Empowering young potential change-makers in Tanzania, Peru and Indonesia | Opinion piece based on a workshop. Primary data is only limited to two paragraphs of individuals expressing their experiences in agriculture. Data is not representative of young people in Tanzania, Peru and Indonesia where the workshop took place. |
| Freije, A. N. et al. (2017) | Preparing Youths for Careers in Agriculture Through State Crop Scouting Competitions | The study was conducted outside of Africa, and the results are not relevant to Africa. The study is on State crop scouting competitions (CSCs), which are contests for high school students that occur annually in each of three states: Indiana, Iowa, and Nebraska. |
| Chipeta, M. E. (2014) | The arithmetic of youth absorption into Africa's farming job market | An opinion piece, no primary data. |
| Castillo-Quero & M. Guerrero-Baena, M. D. (2019) | Structural, productive and financial characterization of farms run by young farmers | The article is written in Spanish. No English version available |
| Adedugbe, A. (2014) | Engaging youths in agriculture through Information and Communications Technology | No primary data. The article is a discussion aiming to identify and highlight the potentials of young Africans in agriculture through Information Communications Technology (ICT) and to arouse the interest of Africa’s youths in sustainable agricultural development in the continent. |
| Akinnifesi, F. K. (2014) | Can South-South Cooperation offer sustainable agriculture-led solutions to youth unemployment in Africa? | An opinion piece, no primary data. |
| Amita et al. (2017) | Entrepreneurial intention of undergraduate students of G. B. Pant University of Agriculture and Technology, Pantnagar | The study was conducted outside of Africa. |
| Bangura, K. S. et al. (2018) | Cultivating young agriculturalists in Kenema, Sierra Leone | Opinion piece based on a workshop. Primary data is only limited to two paragraphs of individuals expressing their experiences in agriculture. Data is not representative of young people in Kenema, Sierra Leone |
| Baulch, B. et al. (2019) | Malawi's challenging employment landscape | Using data from the three national representative household surveys, the paper examines what changes have occurred in the patterns of employment among those of working age in Malawi, focusing on its rapidly growing youth. The study is relevant to the objectives of the research or the inclusion criteria. |
| Bojang, F. (2014) | Special Issue: African youth in agriculture, natural resources and rural development | An opinion piece, no primary data. |
| FAO (2013) | FAO African Youth in Agriculture | Individual articles included in this journal have already been screened |
| FAO (2018) | The rural youth mobility project | The study is based on youth migration patterns in Tunisia and Ethiopia. The variable being investigated is not aligned with the inclusion criteria. |
| FAO et al. (2014) | Youth and agriculture | Each case study is focused on one young person from different countries, in terms of each challenge or limitation identified. This does not give a proper representation of the population. |
| Mutua et al. 2017 | IDS Africa’s youth employment challenge / Youth Participation in Smallholder Livestock Production and Marketing | Duplicate article published in a different journal (Institute of Development Studies) |
| White B. (2017) | IDS Africa’s youth employment challenge/Agriculture and the Generation Problem: Rural Youth, Employment and the Future of Farming |  |
| Anyidoho et al. (2017) | Perceptions and Aspirations: A Case Study of Young People in Ghana’s Cocoa Sector |  |
| Institute of Development Studies (2018) | Stimulating Agribusiness Entrepreneurship to Solve Youth Unemployment in Kenya | Policy brief article. No primary data or clear description of the age range of youth being referred to. |
| Future agricultures (2016)/IDS | The New Alliance on Food Security and Nutrition: What are the Implications for Africa’s Youth? |  |
| Sumberg,J. and Okali, C. (2013) | Young People, Agriculture, and Transformation in Rural Africa: An “Opportunity Space” Approach | Duplicate article published in a different journal (Institute of Development Studies) |
| NEPAD (2019) | 3rd Africa Climate Smart Agriculture Alliance | Concept note, no primary data. |
| Amichi, H. et al. (2015) | A generation looking for opportunities and recognition: young rural people and their pathways into irrigated agriculture in North Africa | The article is written in French. No English version available. |
| Balarubini, M. et al. (2017) | Are rural youth aspiring for agriculture as their avocation? | The research was conducted in India, and the results are not relevant to Africa. |
| Bezu, S. & Holden, S. (2014) | Are rural youth in Ethiopia abandoning agriculture? | The study is based on youth migration patterns in Ethiopia. |
| Bidogeza, J. C. & Kaufmann, R. von (2013) | Realizing the potential of youth in the development of sustainable grasslands | Paper prepared for a conference. No primary data. |
| Brown, M. et al. (2011) | Reclaiming agriculture for Pacific youth | An opinion piece, no primary data. |
| Charette, D. & A.A. Temu (2014) | Using internships to stimulate agricultural entrepreneurship and innovation by graduates of tertiary agriculture education | This is a research Application Summary—no primary data. |
| D'Silva, J. L. et al. (2010) | Socio-demography factors that influence youth attitude towards contract farming | The study was conducted outside of Africa. |
| Getnet et.al (2014) | Becoming a young farmer in Ethiopia: processes and challenges | An opinion piece, no primary data. |
| Lambon-Quayefio, M. (2014) | Youth engagement in agricultural policy processes in Malawi | The study includes youth and other members of the household. |
| MacNeil, C. et al. (2014) | Youth livelihood strategies and environmental decision-making in the Congo Basin forest: the case of southeastern Cameroon | Master's dissertation |
| Thebe, V. (2018) | Youth, agriculture and land reform in Zimbabwe: experiences from a communal area and resettlement scheme in semi-arid Matabeleland, Zimbabwe | The paper is about the land reform problem, focusing mainly on the three decades of land reform in Zimbabwe. The variables being investigated is not aligned with the inclusion criteria. |
| Ricker-Gilbert, J. & Chamberlin, J. (2018) | Transaction costs, land rental markets, and their impact on youth access to agriculture in Tanzania | The variables being investigated are not aligned with the inclusion criteria. |
| Sanagorski, L. A. (2011) | The starting point: youth's perceptions about sustainable agriculture 8-9 | An opinion piece, no primary data. |
| Parkinson, N. (2014) | Back-to-school farming initiative in Liberia: leading student farmers back to the land and into the classroom | Opinion piece based on a workshop. |
| Minde, I. et al. (2015) | Challenges for agricultural education and training (AET) institutions in preparing growing student populations for productive careers in the agri-food system | The article has been removed from the website. |
| Norsida, M (2012) | Unleashing youth potentials in developing the agricultural sector | The article has been removed from the website. |
| Parkinson, N. (2014) | Back-to-school farming initiative in Liberia: leading student farmers back to the land and into the classroom | The article has been removed from the website. |
| Fifolt, M. et al. (2018) | Promoting School Connectedness Among Minority Youth Through Experience-Based Urban Farming | The research was conducted in America, and the results are not relevant to Africa |
| Haggblade et.al (2015) | Motivating and preparing African  youth for successful careers  in agribusiness  Insights from agricultural role models | An opinion piece, no primary data. This paper reports the results of interviews across the continent with distinguished agricultural professionals whom we asked to reflect on their career trajectories and on the many structural changes underway in African agricultural systems. |
| Opara, U. L. (2013) | Perspective: the role of universities in transforming African agriculture for economic development - producing knowledge farmers and entrepreneurial leaders | This paper speaks about repositioning African universities to address challenges and contribute to the economic transformation and well-being of our people. The variables being investigated are not aligned with the inclusion criteria. |
| Waswa, F. (2018) | Perspectives of exiting undergraduate agriculture students on the status of agricultural extension in selected counties in Kenya | The article has been removed from the website. |
| Nhamo, N. & Chikoye, D. (2018) | Models supporting the engagement of the youth in smart agricultural enterprises | No access to the article content and no response from authors. |
| Hasun, R. H. (2015) | Suggested model for rural training youth in the area of farm management | No access to the article and no response from authors. |
| Adelodun OB (2015) | Participation of Youth in Aquaculture | No primary data. The paper is a discussion contributing to the broader literature. |

Table Si 4: Characteristics of studies included in the review

| **Author and year** | **Title** | **Location of the study** | **Study aims** | **Context** | **Study design** | **Sample size** | **The main occupation of the study participants** | **Study outcomes** |
| --- | --- | --- | --- | --- | --- | --- | --- | --- |
| Auta, S.J. et al. (2010) | Rural Youths' Participation in Agriculture: Prospects, Challenges and the Implications for Policy in Nigeria | Nigeria | To evaluate rural youth participation in agriculture and their access to agricultural production resources and services in their localities; To evaluate the effect of youths' access to input and services on-farm productivity and personal welfare;  To recommend agricultural policies intended to assist rural youths in Nigeria. | Rural | Qualitative study | 108 | Farming | Little progress has so far been achieved towards raising the income and living standards of youths engaged in farming. Other challenges being faced by youths engaged in farming include lack of finance, poor access to farm inputs, proper market channels and other services. |
| Adelakun, O.J et al. (2019) | Attitude of Graduate Youths Towards Agro-allied Skill Acquisition and Entrepreneurship Development Programme in Oyo State | Oyo State Nigeria | This study assessed the attitude of graduate youths towards SAED in selected local government areas of Oyo State, Nigeria. | Rural | Qualitative study | 82 | Agriculture (livestock rearing 79.3%, agricultural engineering 20.7%) | Graduate youths had a favourable attitude towards agro-allied skill set of the skill acquisition and entrepreneurship development programme, which is an indication of youths' cognizance of the importance of the programme. However, significant challenges faced by graduate youths in the programme include inadequate supervision of the NYSC officials and lack of sufficient facilities for training. |
| Adeogun S.O (2015) | Participatory diagnostic survey of constraints to youth involvement in cocoa production in the Cross River state of Nigeria | Cross river state, Nigeria | To diagnose the constraints hindering youth involvement in cocoa production in Cross River State, Nigeria | Rural | Qualitative study | 135 | Not mentioned | In this study, it was revealed that many constraints were responsible for youths" reluctance to be involved in cocoa production. Notable among them were non-availability of basic amenities, rigorous nature of cocoa, youth rural-urban migration and youths" involvement in Okada "motorcycle business". This suggests that attention should be given to these constraints to enhance youth involvement in cocoa production in Nigeria. |
| Adesina T.K. & Favour, E. (2016) | Determinants of Participation in Youth-in-Agriculture Programme in Ondo State, Nigeria | Ondo State, Nigeria | To identify the determinants of participation in YIAP | Peri-Urban | Qualitative study | 128 | Not mentioned | YAIP could be a means of introducing new techniques and practices in crop production, properly organized youth in agriculture programmes may help improve the income of youths and active participation in agriculture programmes could raise the production efficiency and productivity of the beneficiaries. |
| Agboola A. F. et.al (2015) | Assessment of youth participation in indigenous farm practices of vegetable production in Oyo State, Nigeria | Oyo State, Nigeria | Assesses youth participation in indigenous farm practices of vegetable production in Oyo State, Nigeria | Rural | Qualitative study | 200 | Farming | There were significant correlations between the level of participation of youth towards indigenous farm practices in vegetable production and the following variables; educational level, household size, farm size, farming experience, income, membership of association, openness, visibility, resources availability, knowledge transferability and community belief system of these indigenous farm practices were also significantly related with the level of participation of youth in indigenous farm practices. |
| Ampadu-Ameyaw R. (2015) | Understanding Farming Career Decision Influencers: Experiences of Some Youth in Rural Manya Krobo, Ghana | Manya Krobo, Ghana | Explores the role of socialization in career choices and attempts to find the link between the aspiration of youth and career outcomes | Rural | Qualitative study | 35 | Not mentioned | Farming aspirations were cultivated/ instilled in these individuals from their childhoods yet not all of them automatically became farmers. The kind of barriers and supports they faced in life as they grew and also made career choices played critical roles in determining what they are currently doing farming. |
| Amsler, K. et al. (2017) | Youth decision making in agricultural adaptation to climate change: an analysis in East Africa | East Africa | To enhance the understanding of youth-specific needs as agricultural production practices shift to incorporate climate-smart techniques, as well as examine the extent to which youth have decision making power in regards to agricultural adaptations to climate change. | Rural | Qualitative study | 164 | Not mentioned | Our findings indicate that the primary barrier for youth implementation of adaptation measures is the limited accessibility of agricultural inputs such as land, water, financial capital, and markets. The barrier is not knowledge-based, as young farmers are already actively adapting to changes in their environment mainly due to the training and education they have received in regards to how to adapt to their changing environment. |
| Ayinde J.O. et al. (2016) | Perception of youths on government agricultural development programmes in Osun state Nigeria | Osun State, Nigeria | To assess the Osun State Rural Enterprise and Agricultural Programme (OREAP) among youths in Osun State, Nigeria. | Rural | Qualitative study | 113 | Trading | The study concluded that the objectives of OREAP such as to train youth to become a good farmer and entrepreneur, to boost agricultural production in the State of Osun was achieved to a reasonable extent in training conducted in 2013. The participation of the youth in the programme is greatly affected by how conducive the environment of the training centres is. |
| Ayinde, J.O. et al. (2015) | Assessment of rural youth involvement in the usage of Information and Communication Technologies (ICTs) among farmers' in Osun State, Nigeria | Osun state Nigeria | The study seeks to assess rural youth involvement in the use of information and communication technologies (ICTs) in agriculture in Osun State, Nigeria. | Rural | Qualitative study | 120 | Not mentioned | The use of ICTs in farming could bring about high productivity if the youths and other agricultural development stakeholders perceive ICTs tools as media of agricultural transformation. Furthermore, in order to enhance paradigm shift in the agricultural practices among our farmers to forestall food insecurity, youth training and retraining in the use of ICTs is highly essential. |
| Ayinde, J.O. et al., (2018) | Assessment of rural youth adoption of cassava production technologies in Southwestern Nigeria | Southwestern Nigeria | To assess rural youths adoption of cassava production technologies by identifying and determining the awareness of cassava production technologies available to the youths and to determine the level of adoption of these technologies among these youth. | Rural | Qualitative study | 135 | Cassava production | The study concluded that farming activities are still male-dominated, . |
| Bagson E. & B.A Naamwintome (2013) | Youth in agriculture: Prospects and challenges in the Sissala area of Ghana | Sissala area, Ghana | To the extent to which the youth participate/are into agriculture, the challenges militating against their participation and the way forward in the study area of the Upper West Region (UWR). | Rural | Descriptive and quantitative survey research design | 185 | Not mentioned | More youths in the study area are without formal education, and this has been attributed to the youth's desire to attain wealth since this serves as a disincentive to attaining formal education. |
| Bello, M et al. (2018) | Secondary School Students Perception of Fisheries as a Profession in Borno State, Nigeria | Borno State, Nigeria | To determine the involvement of secondary school students in harnessing sustainability in fisheries development | Urban | Qualitative study | 280 | Senior secondary school pupils | The perception of secondary school students to fisheries and the willingness to practice fish farming is encouraging, so there is the prospect for fisheries as a profession in Borno state. |
| Cheteni P. (2016) | Youth Participation in Agriculture in the Nkonkobe District Municipality, South Africa | Nkonkobe Municipality, Eastern Cape Province of South Africa. | To assess factors influencing the participation of youth in Agriculture in Nkonkobe District Municipality in the Eastern Cape Province, South Africa. | Rural | A cross-sectional study design | 140 | Temporary employed | Youth have a negative perception of agriculture. Many young people still view agriculture as working in farms physical. Furthermore, several incentives are needed to convince youths that agriculture can provide a good career. |
| Chinsing B. & Chasukwa S. (2012) | Youth, Agriculture and Land Grabs in Malawi | Zomba and Mangochi districts, Southern Malawi | To explores young people's engagement in the agri-food sector in Malawi against the backdrop of two vital related developments, namely: the Farm Input Subsidy Programme (FISP) and the Green Belt Initiative (GBI). | Rural | Not mentioned | Not mentioned | Not mentioned | Young people do not see working in the agricultural sector as a viable means of realizing their dreams, which they link instead to employment in urban areas, engagement in non- farm business enterprises and migration to South Africa to do casual labour. |
| Falola, A. et.al (2013) | Economic Analysis of Rice Production among the Youths in Kwara State, Nigeria | Kwara State, Nigeria | To assess the economic performance of youths in rice production in Kwara State, Nigeria. | Peri-Urban | Qualitative study | 120 | Crop production | The major problems faced by the youth farmers are inadequate credit facilities, lack of knowledge on rice farming, low output price, insufficient land, high cost of inputs and transportation problems. All these problems need to be addressed in order to encourage the youth to actively engage in rice farming as a means of gainful employment opportunity. |
| Felicia W.I et al. (2016) | ASSESSING THE FUTURE OF AGRICULTURE IN THE HANDS OF RURAL YOUTH IN ORIADE LOCAL GOVERNMENT AREA OF OSUN STATE, NIGERIA | Oriade local government Osun state, Nigeria | To assess the Future of Agriculture in the hands of Rural Youths in Oriade Local Government Area of Osun State, Nigeria. | Rural | Qualitative study | 120 | Not mentioned | Age, sex, occupation, level of education and availability of other jobs have a significant relationship with the level of respondents' perception of agriculture as a profession. |
| Hamidu, K. (2015) | Entrepreneurship Intention and Involvement in Agribusiness Enterprise among Youths in Gombe Metropolis, Gombe State, Nigeria: Potentials of Agribusiness in Nigeria | Gombe State, Nigeria | The main objective of the study is to examine the entrepreneurship intention and involvement in agribusiness enterprise among youths in Gombe metropolis, Gombe state. | Peri-Urban | Qualitative study | 440 | Not mentioned | Youth in Gombe metropolis in Gombe State was interested in agribusiness in both livestock and crop production and marketing. |
| Kidido, J.K et al. (2016) | Dynamics of youth access to agricultural land under the customary tenure regime in the Techiman traditional area of Ghana | Techiman traditional area, Ghana | To examine the dynamics of youth access to agricultural land under the customary tenure regime in the Techiman Traditional Area (TTA) to improve on youth access to agricultural land in Ghana. | Rural | Qualitative study | 455 | Farming | Youth land ownership is limited, and access equally constrained under the customary tenure regime in the TTA. The ability of youth to access viable land sizes and on favourable terms is mediated by many variables including cost and socio-cultural factors. |
| Mukembo C.S et al. (2015) | Intentions of Young Farmers Club (YFC) Members to Pursue Career Preparation in Agriculture: The Case of Uganda | Uganda | To determine the perceptions of members of YFCs on their intent to pursue agriculture-related career preparation after graduating from secondary school, including indications of familial support for previous experiences. | Urban | Qualitative study | 102 | Secondary school pupils | A majority of students irrespective of sex indicated their parents were supportive of them participating in the YFCs. This may imply the students' parents valued the activities of the clubs and saw them as avenues that could provide opportunities for the personal growth and development of their children, whether male or female. |
| Ntshangase, W. M., et al. (2016) | The sustainability of small scale cane growers through involvement in the North Coast region of KwaZulu-Natal South Africa | KwaZulu-Natal, South Africa | To understand whether the heirs of small scale cane growers are currently involved in farming activities and whether current participation will increase their willingness to succeed their parents as cane farmers. | Rural | Qualitative study | 193 | Not mentioned | The assumption was that there is minimal involvement or total lack of youth involvement in farming activities. It was worthy to note, from the results, that in addition to respondents’ personal involvement, their siblings and other youth in the study area were also involved in farming. Furthermore, respondents claimed to know other young people, outside of their families, who are involved in farming activities. |
| Nwankwo, O. C.(2014) | Major Factors Militating against Youths Participation in Agricultural Production in Ohafia Local Government Area of Abia State, Nigeria | Abia State, Nigeria | To isolate the major factors militating against youth participation in agricultural production in Ohafia Local Government Area of Abia State, Nigeria. | Rural | Qualitative study | 194 | Students | The youths are not satisfied to be farm labourers, renters and partners. Low performance of the youths affects the food production in Ohafia Local Government Area of Abia State. |
| Okon D.P & Okon Nsa, S (2012) | Determinants of Sustained Youth Participation in Fishing in Mbo Local Government Area of Akwa Ibom State, Nigeria | Akwa Ibom State, Nigeria | To examine the determinants of youth participation in fishing and fishing activities in the study area to identify possible factors that require improvement. | Peri-Urban | Qualitative study | 128 | Fishing | Meeting the sustainable needs of fishing youth has become a significant factor in enhancing food security in the country. Therefore, to meet the rapidly growing protein food demand and hence raise rural income, underlying social and infrastructural facilities must be provided in fishing areas to increase productivity. |
| Sumberg J. et al. (2017) | Young people’s perspectives on farming in Ghana: a Q study | Tepa, Ghana | To report the perspectives of student's attitude toward farming in two high schools in rural Ghana | Urban | Q Methodology | 38 | All participants were students | The explanation of young people's attitude toward farming is more to do with young people's sense of themselves than anything particular about farming. They want new jobs; have more education than their parents. |
| Trevor S. & Kenya J.M (2018) | Rural Youth Participation in Agriculture in Zambia | Kitwe district Zambia | To gain an understanding of the significance and constraints associated with rural youths’ involvement in agriculture in Zambia using a case study of Kitwe district. | Peri-Urban | Qualitative study | 80 | Not mentioned | Rural youth involvement in agriculture is characterized with constraints that include lack of access to capital, inadequate storage facilities, sparse road networks, poor access to agriculture insurance for farm produce, and lack of technical assistance. |
| Yunusa, P. M & Giroh, D. Y. (2017) | Determinants of Youth Participation in Food Crops Production in Song Local Government Area of Adamawa State, Nigeria. | Adamawa State, Nigeria | To describe the socio-economic characteristics of youth in the study area; examine determinants of youth participation in food crops production and identify constraints faced by respondents in food crops production in the study area. | Rural | Qualitative study | 121 | Not mentioned | There was a moderate level of participation of youth in food crops production in the study area that were mostly male. Marital status, educational level, farm size and income were the main determinants of youth participation in food crops production. |

Table Si 5: Policies mentioned in included studies

| **Author** | **Policies mentioned in the study** | **Reference in study** |
| --- | --- | --- |
| Auta, S.J. et al. (2010) | Young Farmers Club (YFCs) | Rural youths targeted projects and programmes. |
|  | National Directorate of Employment (NDE) | Failure of programme to make a significant impact |
| Adelakun, O.J et al. (2019) | National Directorate of Employment (NDE) initiated 1986, Youth Enterprise with Innovation in Nigeria (YOUWIN) initiated 2011 | Achieve youth independence and improved economic status |
| Adeogun S.O (2015) | National Directorate of Employment | To stimulate youth interest in agricultural production, processing and to provide vocational training to the youth. |
|  | Better Life Programme (1987) | To empower women, especially female youths in rural areas through skills acquisition and healthcare training. |
|  | The Fadama programme | To enhance food self-sufficiency, reduce poverty, and create opportunities for employment for youths in rural areas |
| Adesina T.K. & Favour, E. (2016) | Youth in Agriculture Programme (YIAP) | To enhance the involvement of youths in agriculture. |
|  | Wealth Creation Agency (WECA) and New Generation Farmers (NGF) | Create employment for the youth through active participation in modern agricultural practices. |
| Amsler, K. et al. (2017) | Kenyan National Youth Policy | The National Youth Council is responsible for coordinating youth activities and supporting youth policy. |
|  | Tanzania National Youth Policy | To prioritize employment opportunities and social security for youth. |
| Ayinde, J.O. et al., (2018) | Agricultural Transformation Agenda | Enhancing the role of agriculture as an engine of inclusive growth leading to rural employment, wealth creation, and diversification of the economy |
| Bagson E. & B.A Naamwintome (2013) | Comprehensive Africa Agriculture Development Programme (CAADP) and the New Partnership for African Development (NEPAD) programme | To raise the amount and quality of food produced in Africa; that is, secure supply of food for families, communities and countries as well as making export more profitable. |
| Cheteni P. (2016) | Land Redistribution programme | Redistribution of the land taken during apartheid to black farmers. |
|  | Reconstruction and Development Programme (RDP) | Address unemployment, inequality and economic growth. |
|  | Accelerated and Shared Growth Initiative | Creation of employment opportunities for youth |
|  | Growth (AsgiSA), Employment Redistribution Strategy (GEAR) |  |
| Chinsing B. & Chasukwa S. (2012) | Farm Input Subsidy Programme (FISP) | GBI to secure the long-term food security and sufficiency gains through the development of small and large- scale irrigation schemes and maximization of rain-fed agriculture practice. |
|  | The Green Belt Initiative (GBI), | We are prioritizing the agricultural sector as the engine of growth and poverty reduction. |
|  | Poverty Alleviation Programme (PAP) (1995) |  |
|  | Malawi Poverty Reduction Strategy (MPRS) (2001) |  |
|  | Malawi Growth and Development Strategy (MGDS) (2006) |  |
|  | The Malawi Rural Development Fund (MARDEF) |  |
|  | Youth Enterprise Development Fund (YEDEF). |  |
| Falola, A. et.al (2013) | Children-In- Agriculture Programme (CIAP) | Cognizance of the circumstances of poverty, unemployment and deprivations that are prevalent in Nigeria and some other developing countries |
| Kidido, J.K et al. (2016) | National Youth Policy of Ghana and the National Land Policy document | To promote youth involvement in agriculture as a career through the provision of resources for the participation of the youth in modern agriculture. |
